# A Comparison of Tools Used for Tuberculosis Diagnosis in Resource-Limited Settings: A Case Study at Mubende Referral Hospital, Uganda

**DOI:** 10.1371/journal.pone.0100720

**Published:** 2014-06-26

**Authors:** Adrian Muwonge, Sydney Malama, Barend M. de C. Bronsvoort, Demelash Biffa, Willy Ssengooba, Eystein Skjerve

**Affiliations:** 1 Department of Food Safety and Infection Biology, Centre for Epidemiology and Biostatistics, Norwegian University of Life Sciences, Oslo, Norway; 2 The Roslin Institute, College of Medicine and Veterinary Medicine, University of Edinburgh, Easter Bush, Midlothian, Edinburgh, United Kingdom; 3 Institute of Economic and Social Research University of Zambia, Lusaka, Zambia; 4 College of Medicine, University of Arizona, Tucson, Arizona, United States of America; 5 Mycobacteriology Laboratory, Department of Medical Microbiology, Makerere University College of Health Sciences, Kampala, Uganda; University of Ottawa, Canada

## Abstract

**Background:**

This study compared TB diagnostic tools and estimated levels of misdiagnosis in a resource-limited setting. Furthermore, we estimated the diagnostic utility of three-TB-associated predictors in an algorithm with and without Direct Ziehl-Neelsen (DZM).

**Materials and Methods:**

Data was obtained from a cross-sectional study in 2011 conducted at Mubende regional referral hospital in Uganda. An individual was included if they presented with a two weeks persistent cough and or lymphadenitis/abscess. 344 samples were analyzed on DZM in Mubende and compared to duplicates analyzed on direct fluorescent microscopy (DFM), growth on solid and liquid media at Makerere University. Clinical variables from a questionnaire and DZM were used to predict TB status in multivariable logistic and Cox proportional hazard models, while optimization and visualization was done with receiver operating characteristics curve and algorithm-charts in Stata, R and Lucid-Charts respectively.

**Results:**

DZM had a sensitivity and specificity of 36.4% (95% CI = 24.9–49.1) and 97.1%(95% CI = 94.4–98.7) compared to DFM which had a sensitivity and specificity of 80.3%(95% CI = 68.7–89.1) and 97.1%(95% CI = 94.4–98.7) respectively. DZM false negative results were associated with patient’s HIV status, tobacco smoking and extra-pulmonary tuberculosis. One of the false negative cases was infected with multi drug resistant TB (MDR). The three-predictor screening algorithm with and without DZM classified 50% and 33% of the true cases respectively, while the adjusted algorithm with DZM classified 78% of the true cases.

**Conclusion:**

The study supports the concern that using DZM alone risks missing majority of TB cases, in this case we found nearly 60%, of who one was an MDR case. Although adopting DFM would reduce this proportion to 19%, the use of a three-predictor screening algorithm together with DZM was almost as good as DFM alone. It’s utility is whoever subject to HIV screening all TB suspects.

## Introduction

Tuberculosis (TB) remains a disease of major global public health concern in spite of the efforts to combat it. The WHO estimates that about three million people who developed TB in 2012 were missed by the national notification systems [1]. Experts in the field emphasize that a successful international and national TB control program ought to be hinged on accurate diagnosis of active TB cases [2], [3]. Although case detection in developing countries likes Uganda has increased over the years, some resource limited settings like Mubende have continued to register poor case detection levels [4]. There are various TB diagnostic tools available [5], however, there is a universal recognition of limitations with the commonly used tools.

Direct smear microscopy (DZM) which uses Ziehl-Neelsen as the acid-fast dye is the most commonly used of the WHO recommended diagnostic tool for tuberculosis in low and middle-income countries [3], [6]. This is mainly because it is cheap, easy to operate and rapid [6]. However, the test has a low sensitivity, with a detection limit of between 5,000–10,000 bacilli/ml of sample [7]–[9]. For patients with a low bacillary load, as seen in HIV patients and infants, true positive cases can easily be missed [10]–[12]. It has been suggested that the low sensitivity of DZM could partly be due to the fact that facilities in high incidence areas where the test is used as a sole diagnostic tool are usually resource limited, and thus leading to high workload on personnel [10]–[12]. Over the years there have been better diagnostic techniques, however their adaptation to resource poor settings remains a challenge. For example the low sensitivity of DZM can be improved by sputum concentration and/or use of direct fluorescence microscopy (DFM) which uses auramines as the acid-fast dye [13], [14]. This test has an added advantage of requiring only one or two sputum specimens rather than three to reach an acceptable level of performance, which in effect reduces the diagnostic time [13], [14].

Several studies documenting the diagnostic gain of DFM have recommended its implementation [5], [13]. Unfortunately, most of these studies have been performed at validation stages and only a few have documented its performance in routine practice. It is therefore important to document the performance of DFM compared to DZM in a resource limited clinical setting so as to inform policy makers on proper implementation of TB diagnostics [14].

Culture of mycobacteria on solid media has been considered the gold standard for tuberculosis diagnosis. The minimum detection limit of 10 mycobacteria/ml significantly surpasses the minimum detection limit given by DZM [15]. Unfortunately, the growth of TB bacilli requires a protracted period of 4–8 weeks, delaying appropriate treatment in the absence of a confirmed diagnosis. The Mycobacteria growth indicator tubes (MGIT) on the other hand is a fully automated, non-invasive system for recovery of mycobacteria in liquid culture medium but has a draw-back of being expensive [16]. Previous laboratory studies have shown a higher and faster recovery of mycobacteria from sputum specimens than culture on solid medium [17], [18] but the crucial diagnostic value is better harnessed if deployed in large diagnostic facilities. This is because of the infrastructure, high skill and maintenance requirements all of which are scarce in rural settings.

It is noteworthy that, under and over diagnosis contributes to further spread of the disease and wastage of scarce resources on inappropriate treatment respectively. It is therefore crucial to find a cost effective sequences and/or combination of these tools to strike a balance between these two attributes. The use of an algorithm/screening-sequence that combines both the clinical picture and diagnostic tools could guide physicians in establishing this balance. There have been multitudes of conflicting evaluation reports on the efficiency of the 2007 WHO diagnostic algorithms for TB diagnosis in different settings [8], [19]–[21].

Therefore it is critical to continuously refine diagnostic protocols in resource-limited settings. This will not only improve case detection, but also identify areas within the diagnostic chain that are most likely to lead to over and under diagnosis (misdiagnosis).

Therefore the aim of this study was to compare diagnostic tools used at the Mubende regional referral hospital with an extra set of tools used during a research study period in order to:

- Assess the performance of direct Z&N microscopy (DZM) in detecting TB cases from clinical specimens.
- Estimate the utility of three TB-associated predictors in an algorithm with and without DZM.

To achieve these, results from direct smear microscopic examination of sputum, lymph node and abscess aspirates at Mubende regional referral hospital were compared to results from duplicates analysed using fluorescent microscopy (DZM), culture on LJ media and MGIT at the Mycobacteriology Laboratory, Department of Medical Microbiology, Makerere University [22], [23]. Only TB cases caused by bacteria in the *Mycobacterium tuberculosis* Complex (MTC) were considered in this analysis [22], [23]. It should also be noted that DZM is the only diagnostic tool used at Mubende regional referral hospital. Although the hospital has radiology facilities, these are mainly used for orthopaedic purposes.

## Materials and Methods

### Ethical considerations

Full ethical clearance (ref: HS 879) was obtained from the Uganda National Council for Science and Technology (UNCST). Prior to this study, healthcare authorities and the research team were briefed about the ethical issues. Due to logistical and facility setup, oral consent was obtained from participating patients (documented on the information sheets), something that was in line with the research ethical mandate given by UNCST. Furthermore, data were anonymised before analyses as stipulated by the UNCST guidelines of research involving human as research participants (2.2/b-e/2007).

### Study site

Mubende district is located in the central region of Uganda, inhabited by approximately 750,000 people of whom an estimated 64% live below the poverty line in population dense urban and peri-urban areas [24]. Piot’s model on TB treatment and health seeking suggests that only 20% of examined individuals seeking care at a health facility will be diagnosed [25]. In this regard, according to socio- anthropological studies on mycobacterial infections in the Uganda [26], it is estimated that only 35% of the Ugandan population, especially in the Uganda cattle corridor (where Mubende districts is located), seeks health care from allopathic practitioners [26]. This status quo would suggests that only a fraction of TB cases make it to hospitals which probably explains the low TB case detection level of 37% in Mubende district [4].

### Study design and population

This study was part of a wider study of the molecular epidemiology of TB in Mubende district of Uganda conducted by the same authors and described elsewhere [22], [23]. Briefly, data was obtained from a cross sectional study conducted between February and July 2011. The participating patients had to have presented with cervical lymphadenitis/abscesses and/or a cough that had persisted for at least two weeks at Mubende regional referral hospital. The national tuberculosis program (NTP) is only run at this referral hospital in the Mubende district and all persistent ailments are treated here. This implies that the majority of residents in this district who met the study’s criteria would most likely be treated at this facility.

### Sample collection

**Figure 1:**
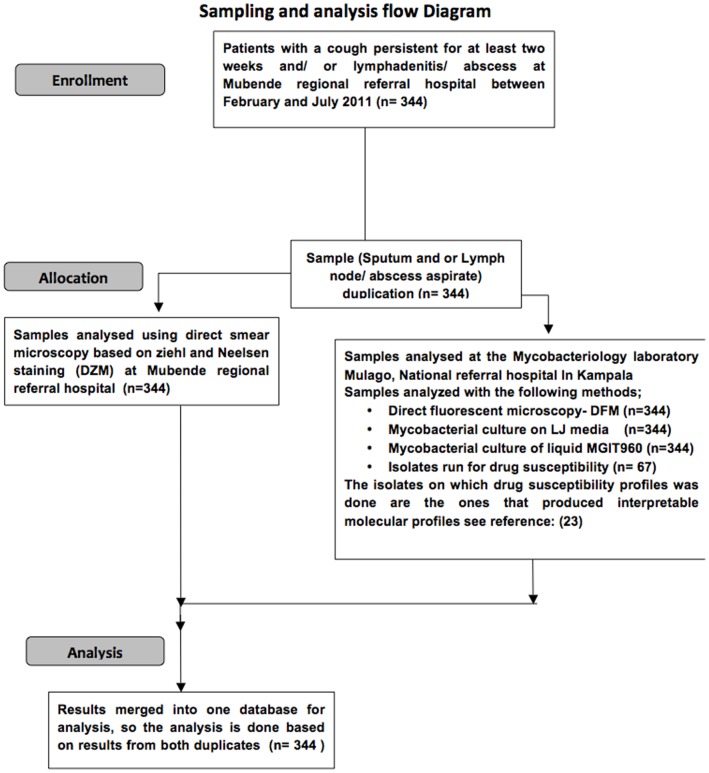
Sampling and sample analysis flow chat.

Sputum and/or lymph node aspirates were collected using standard procedures as described by Muwonge *et al*. [23]. One appropriate sample was collected from patients that had either a cough or lymphadenitis/abscess, or both from patients that had both clinical presentations. A total of 344 samples from 344 patients were taken in duplicates. One of the duplicates was delivered to the diagnostic laboratory within Mubende regional referral hospital for DZM and the other was delivered to the bio-safety level 3 (BSL3) Mycobacteriology Laboratory, Department of Medical Microbiology, Makerere University. Here direct fluorescent microscopy (DFM), culture and drug resistance tests (DST) were carried out (Figure 1). A questionnaire was also administered to each patient to retrieve information about the patients’ socio-economic welfare, as an addition to the clinical information (Table S1).

### Diagnostic Tools

#### Direct Ziehl-Neelsen smear microscopy (DZM)

At Mubende regional referral hospital, direct smears from each sample were examined with the Ziehl-Neelsen **(**ZN) method (Figure 1). A sample smear prepared on slide, air-dried and heat fixed. It was flooded with 1% carbol fuchsin and heated (steaming NOT boiling) for five minutes. This was then washed with water and decolorized using 3% acid-alcohol followed by a brief wash with water. It was then stained with 0.3% methylene blue for 30–60 seconds and then washed with water and air-dried. This slide was then visualized under a light microscope (Olympus CX31) using a 10^3^ magnification. A positive sample was defined as one that showed acid-fast rods in a 300 fields of the slide as recommended by WHO [27]–[29].

#### Direct fluorescent smear microscopy (DFM)

Smears were made from the duplicate samples and stained for fluorescent microscopy according to standard method [5], [28]. The light source used was a Light Emitting Diode (LED) (Fraen Corporation Srl, Via delle Querce, Trivolzio(Pv) Italy). Thereafter the bacillary load was established. Bacillary load is a measure of culture forming units as seen with the aid of staining dye and is used to quantify the number of bacilli in a sample [27]–[29]. If a sample contains 10–99 acid fast bacilli (AFB) per 100 fields it is categorized as +1, and if it contains 1–10 AFB/field in 50 fields this is categorized as 2+, while if a sample contains >10 AFB/field in 20 fields it is categorized as 3+ [27], [30].

#### Mycobacterial culture

The sputum samples were processed according to standard procedures [18], [31], inoculated on blood agar, Lowenstein Jensen (LJ) (BD BBLTM; Franklin Lakes, NJ, USA) slants incubated for up to eight weeks and in *Mycobacterium* Growth Indicator Tube (BD BBL MGIT960, Franklin Lakes, NJ, USA) incubated for six weeks as described [22], [23]. The criteria for verification of isolates belonging to the MTC and the drug resistance procedures were as described [5], [18]. Note that the use of blood agar was for contamination detection. Culture contamination was considered if a DZM AFB negative sample grew on blood agar (Table S2).

#### Drug susceptibility profile

The drug susceptibility profiles were obtained from previously published data by the same author for the relevant patients [22], [23]. The susceptibility to four drugs used as first line therapy namely; Streptomycine, Rifampicine, Isoniazid and Ethambutol of DZM false negative cases were identified and used for the public health component of this study.

### Data management and analysis

Information obtained from the questionnaires, HIV status from individual health record and corresponding TB results for each patient were entered in Excel 2007. The data were then exported to Stata (Stata ver. 11/SE for Windows, Stata Corp, College station) and R software version 2.15.3 (http://cran.r-project.org/) for appropriate statistical analyses.

#### Estimation of diagnostic test characteristics and agreement

Assessment of diagnostic characteristic was done using the *diagt* command in Stata (Stata ver. 11/SE for windows, Stata Corp, College station) considering DZM and DFM as the tests to be evaluated and culture on LJ media as the reference test (gold standard). The kappa measure of agreement between diagnostic tests was also calculated.

#### Survival analysis of time to detection of bacilli on MGIT960

Survival analysis was done using the *survival* package in the R software environment, the same excel data set was converted into a CSV file and imported into R for the survival analysis. The time (T = 19 days) denotes the maximum time to detection in this study, but the total time of observation was 6 weeks (42 days). Using the *Surv* function within the survival analysis package an event was defined as becoming positive and time to detection measured in days. Note that the censor time is set at T = 19. Kaplan-Meier graphs were generated for each explanatory variable in the descriptive survival analysis. A Cox proportion hazard model was built from explanatory factors that showed significant variation in time to detection and fulfilled the model assumptions. The post model evaluation was done using standard procedures.

#### Diagnostic/screening algorithm

The diagnostic/screening algorithm in this study is a layout of a logistic regression model that contains some of the WHO recommended predictors [3], [28] **.** The analysis in this study explored the model (algorithm) case prediction in each of the sub-populations formed by the different combinations of clinical and diagnostic aspects so as to give an estimate of the likely-hood of case detection. In order to estimate the utility (predictive ability) of this diagnostic algorithm, features of the clinical history and DZM results were used as predictors of true disease status (culture on LJ results) in a multivariable logistic regression model. The model was built in a forward step-by-step approach that resembles the events leading to a physician requesting for DZM test in Mubende referral hospital. At each step the proportions of true cases and predicted cases (Probability of being a case) are calculated and presented in an algorithm tree/chart. Logit(p*TB*)* = *β_0_+βX_1_+ β_2_X_2_+ β_3_X_3_ *….*

Where pTB is the predicted probability of being TB positive on LJ media, x_1,_ x_2_ and x_3_ are the predictors in the model while β_0_ is the intercept term and β_1_, β_ 2_ and β_3_ are regression coefficients.

All the predictor variables included in the final model gave at least P = 0.25 in the univariable logistic regression. In order to maximize the number of true cases classified when DZM is used in this algorithm, a receiver operating characteristic curves (ROC) analysis was done to identify the lowest probability cut-off with the highest sensitivity and specificity. The final model validation and post estimation analysis were done using the standard statistical methods in Stata.

## Results

344 individuals were examined, 206 (60%) were male and 138 (40%) were female, with an average age of 31.5 (18.5–44.3) years, height of 155.7 (153.8–157.4) cm and weight of 50.4 (49.2–51.55) kg. Forty-seven percent of the patients were married and 41% single, the rest widowed, divorced or too young to be categorized.

### Summary of DZM, DFM and culture results

The descriptive results of DZM, DFM and culture are given in Table 1. Acid-fast bodies (AFB) were identified 32 (9.3%) samples from 344 patients examined by DZM at the Mubende regional referral hospital. Samples from 61 patients (18%) had AFB on DFM, and growth on LJ media detected 66 cases (19%) (Table 1).

**Table 1:** Descriptive results comparing DZM, DFM and culture on LJ for clinical specimens obtained from 344 patients at Mubende referral hospital, Uganda stratified by patient’s age, sex and HIV status as well as type of specimen and bacilli load in specimen.

Patient profile	Label	DZM		DFM		Culture on LJ media	
		+	−	+	−	+	−
Gender	Male	19	187	38	168	47	159
	Female	13	125	23	115	19	119
Age	Young	2	41	2	12	6	37
	Middle aged	29	258	55	235	57	230
	Elder	1	13	4	39	3	11
HIV status	Negative	7	53	13	47	11	49
	Positive	19	63	39	43	42	40
	Unknown	6	196	9	193	13	189
Sample	Sputum	16	162	34	144	38	140
type	LN aspirate	15	130	21	124	19	126
	Abscess aspirate	1	20	15	6	9	12
	Negative	9	278			17	270
Bacillary load	AFB+1	1	7			6	2
	AFB+2	1	3			4	0
	AFB+3	21	24			39	6

Note that there is no result for bacillary load on DFM because the bacillary load was established using DFM. Age category: Young≤20, Middle aged≤55, Elders>5.

Most of the cases detected by DZM had a bacillary load of +3. Of the DZM positive patients, 19 (59.3%) and 13 (40.6%) were male and female respectively. The same trend is reflected throughout the test on the rest of the diagnostic tools. A majority of the DZM positive samples were recovered from HIV infected middle-aged patients. A prospective follow-up of the specimen analysis from DZM through DFM and then culture showed a steady increase in the number of positive cases detected (Table 1). It is noteworthy that the culture contamination proportion was 6/344 or approximately 2% of the cultured samples showed culture contamination (Table S2).

### Diagnostic features and comparison of tests

DZM was able to detect 36.4% of the true TB cases in this study. This test had a sensitivity and specificity of 36.4% (95% CI = 24.9–49.1) and 97.1% (95% CI = 94.4–98.7) when compared to the gold standard respectively. The comparison with DFM as the gold standard against DZM showed that DZM picked up 25 of 61 cases positive on DFM. The low sensitivity of DZM was also reflected in the low kappa agreement measure against the gold standard. It is noteworthy that there was no significant difference in false positives recovered when DZM was compared to the gold standard (Table 2).

**Table 2:** Diagnostic performance of DZM&DFM against culture on LJ (gold standard) in detection of bacilli in clinical samples obtained from patients at Mubende referral hospital, Uganda.

Evaluated test		Gold standard	
	DZM/DFM*	DZM/LJ	DFM/LJ
	Positive Negative	Positive Negative	Positive Negative
Positive	25 7	24 8	53 8
Negative	36 276	42 270	13 270
		Estimate (95%CI)	Estimate (95%CI)
Sensitivity (%)		36.4(24.9–49.1)	80.3(68.7–89.1)
Specificity (%)		97.1(94.4–98.7)	97.1((94.4–98.7)
Positive predictive value (%)		75.1(56.6–88.5)	86.9(75.8–94.2)
Negative predictive value (%)		86.5(82.2–90.1)	95.4(92.3–97.5)
Kappa agreement measure (%)		41.6(36.6–46.6)	79.7(74.3–85.1)

*DFM is used as the gold standard with DZM. Note that the rest of the comparison is done with Results on LJ media as the gold standard. The status (+/−) of the reference tool will be the column status while the test tool is the row. For example (DZM/DFM) = row (+/−)/col(+/−).

DFM detected 53 of the 66 cases detected by the gold standard and therefore had a sensitivity and a specificity of 80.3% (95% CI = 68.7–89.1) and 97.1% (95% CI = 94.4–98.7) respectively. This too is reflected in the high kappa agreement measure against the gold standard. Consequently, DFM produced a higher positive and negative predictive value than DZM (Table 2). It should be noted that although MGIT 960 recovered more positive cases (n = 69) than culture on LJ media (n = 66) (Table S2), given that these two agreed on 60 cases, the latter was considered as the gold standard for this study.

### DZM-false negative cases

A significant proportion of HIV positive cases gave false negative results on DZM. In fact the odds of false negative results among HIV positive patients were six times those of HIV negative patients (Table 3). Smoking also appears to be associated with false negative result, this is because the odds of false negative DZM results among smokers were twice those of non-smokers. Similarly, sample type (clinical presentation) was also associated with false negative results i.e. the odds of an abscess aspirate giving a false negative results was almost six times that of a sputum sample (Table 3). The drug susceptibility profiles of DZM false negative cases showed that eight cases were resistant to at least one drug used in the first line treatment against TB in Mubende district (Table 4). Of these one was resistant to both Rifampicin and Isoniazid, hence classified as multidrug resistant (MDR).

**Table 3:** Shows factors associated with DZM false negative results.

Variable	Level	Odds ratio (95% CI)	p-value
HIV status	Negative	–	–
	Positive	6.53(2.29–18.58)	0.00
	Unknown	0.38(0.12–1.21)	0.10
Tobacco usage	Non smokers	–	–
	Smokers	2.32(1.07–5.01)	0.03
Sample type	Sputum	–	–
	Lymph node aspirate	0.52(0.22–1.26)	0.15
	Abscess aspirate	6.32(1.87–21.33)	<0.01

AUC = 0.84.

**Table 4:** Drug susceptibility profiles for DZM false negative cases at Mubende regional referral hospital, Uganda.

Case number	Sample type	Diagnostic tools			Drug resistance MDR(+/)				
		DFM	MGIT960	LJ	S	E	I	R	MDR-TB
#56	Sputum	+	+	−	r	s	s	s	−
#33	Sputum	+	+	+	r	s	r	r	+
#66	Sputum	+	+	+	r	s	s	s	−
#30	Sputum	+	+	+	r	s	s	s	−
#118	Lymph node aspirate	−	+	+	s	s	r	s	−
#51	Lymph node aspirate	+	+	+	s	s	r	s	−

LJ = Lowenstein-Jensen; S = streptomycin; E = Ethambutol; I = Isoniazid; R = Rifampicin: MDR-TB multi drug resistant Tuberculosis, Drug resistance r = resistant, s = susceptible.

### Evaluation of time to detection on MGIT960

**Figure 2:**
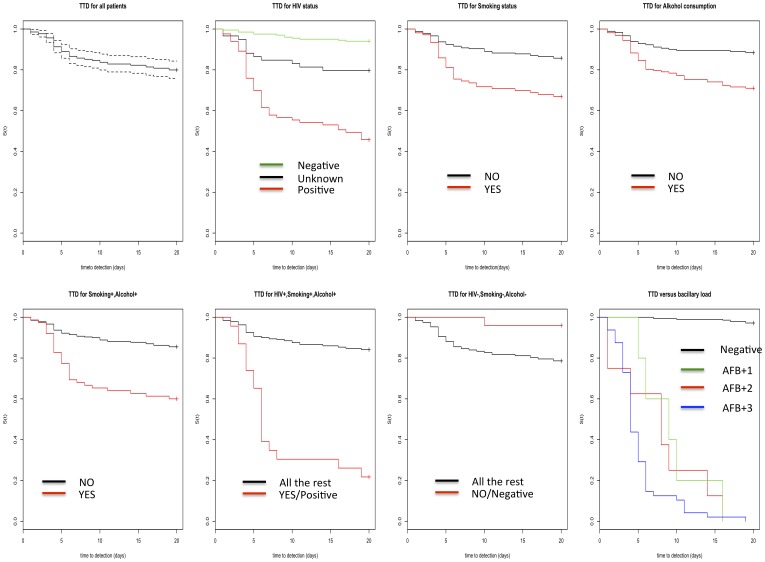
Kaplan-Meier plots showing the Time to detection (TTD) of bacilli on MGIT960 (Y-axis = s(t) which is the survival function, X-axis is the TTD in days). Top row first graph shows the time to detection of all the 344 patients without any covariate structure considered. Second, third and fourth show the TTD when HIV status, Smoking and alcohol consumption are considered as covariates respectively. Bottom row: The first, second, third and fourth graph show the TTD for the combination of (patients who consume alcohol and smoke), (patients who smoke, consume alcohol and are HIV positive), (HIV negative patients who neither consume alcohol nor smoke) and bacillary load levels respectively.

The time to detection, (TTD) of bacilli on MGIT 960 is presented in Figure 2 and the hazard ratios are given in table 5. In general, the majority of patients’ time to detections was 2–4 days, but the maximum time to detection was 19 days. From the Kaplan-Meier plots the time to detection on MGIT960 appears to be a function of bacillary load in a specimen (Figure 2). The Cox proportional hazard model shows that the hazard of becoming a positive culture on MGIT960 conditional on the patient presenting with a cough persistent for at least two weeks and/or lymphadenitis/an abscess is ∼19 times higher if the patient is also HIV positive and ∼7 times higher if they are a smoker or consume alcohol (Table 5).

**Table 5:** Cox proportional hazard model of the time to detection of *M. tuberculosis* bacilli on MGIT960 based on 344 patients at the Mubende referral hospital.

Variable	Level	Coefficients (95% CI)	Hazard Ratio (95% CI)	p-value
HIV status	Negative	–		–
	Positive	2.94 (1.54–5.59)	18.9 (4.66–267)	0.001
	Unknown	0.27 (0.11–0.59)	1.31 (1.12–1.80)	0.001
Tobacco usage	Non smokers	–		–
	Smokers	1.94 (1.19–3.17)	6.96 (3.29–23.8)	0.007
Alcohol usage	Non consumers	–		–
	Consumers	1.88 (1.10–3.20)	6.55 (3.00–24.5)	0.019

### Diagnostic/screening algorithm

**Figure 3:**
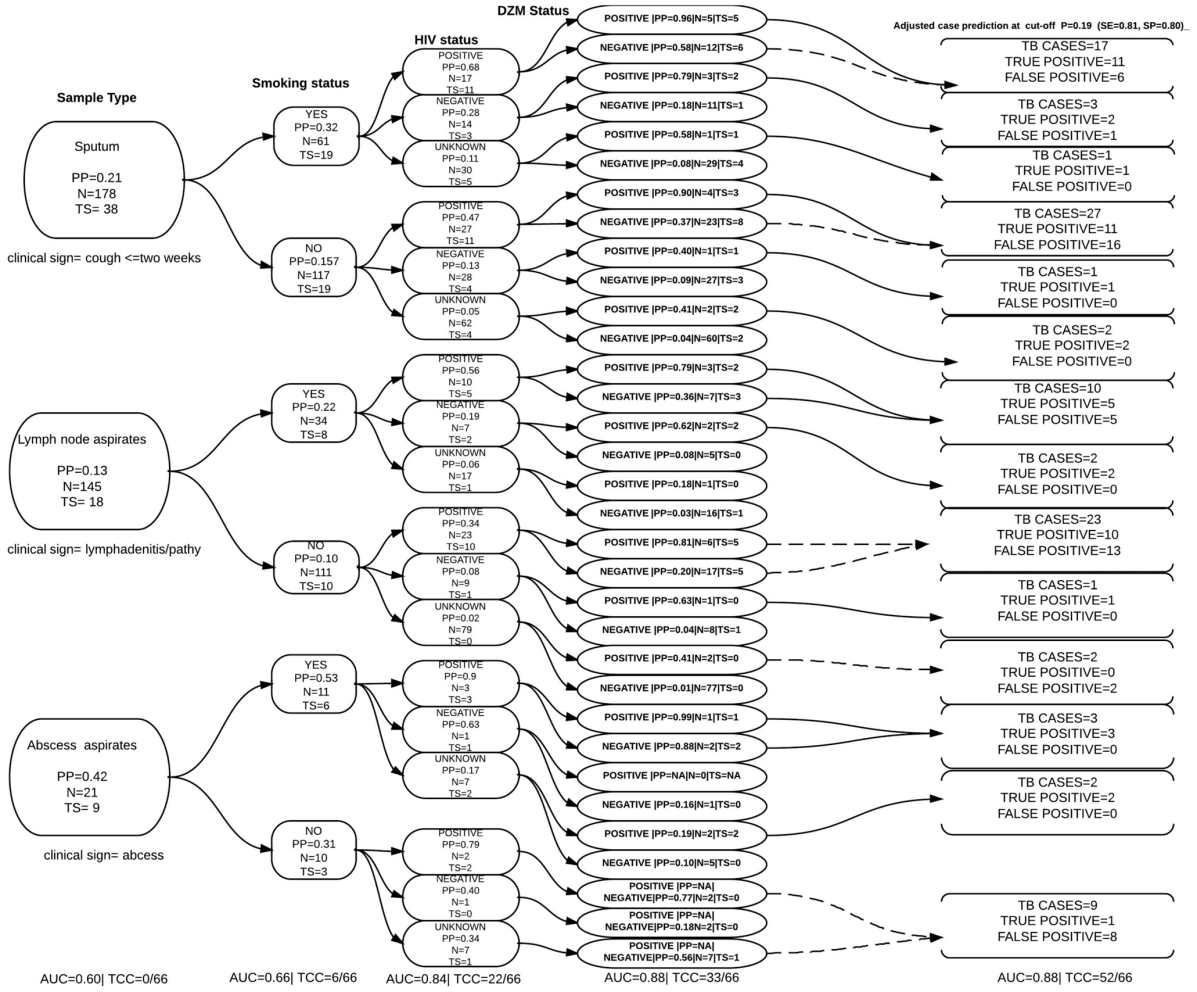
The diagnostic algorithm chart showing the case proportions formed by clinical and diagnostic combinations with data from Mubende regional referral hospital, AUC is the area under the curve, PP = predicted probability of being TB case, TS = total number of true TB cases in each covariate pattern based on the gold standard, N = the total number of individual in a specific covariate pattern (sub-population formed by combining clinical factors), TCC = total number of TB-cases accurately classified my the model. The adjusted case predictions are made at a probability cut off P = 0.199 as established in figure 3, the broken lines shows pathways that are most likely to increase numbers of false positive cases.

**Figure 4:**
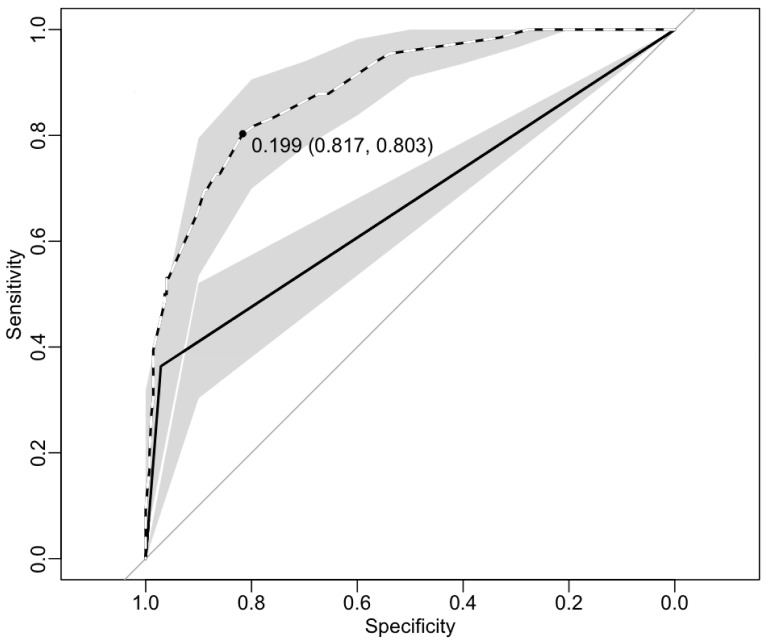
The receiver operating characteristics curve (ROC) showing the lowest cut-off probability with the highest specificity and sensitivity for the diagnostic/screening algorithm, this probability is used to adjust the case classification in figure 2. The Solid line represents DZM on its own at AUC = ∼66%, the dotted line shows the model with DZM and the three predictors at AUC = ∼ 88%.

A patient from whom sputum and/or a lymph node or abscess aspirate was taken must have presented with a cough that had persisted for at least two weeks and/or lymphadenitis or an abscess. This clinical facet alone contained 38, 18 and 9 true TB cases proportion-wise respectively. However if this were to be used alone as predictor in this algorithm (Model), the model would not be able to classify any cases regardless of the high AUC (Figure 3). (The author has chosen to follow HIV positive smokers for the purposes of explaining). When this clinical picture was combined with the patient’s tobacco smoking status, the number of true TB cases among smokers was 19, 8 and 6 respectively. At this step the model would be able to accurately classify 6 of the 66 true cases with AUC = 0.66. If the HIV status of these patients was then added to the model, the total number true cases among HIV positive smokers were 11, 5, and 3 respectively. At this step the model could accurately classify 22 of the 66 true cases. At this point if the physician asked the patient to take a DZM test, the number of true cases among DZM positive, HIV positive smokers was 5, 3 and 1 respectively. At this point the model could accurately classify 33 of the 66 true cases with AUC = 0.88. The optimized probability cut-off with the highest sensitivity and specificity is present in (Figure 4). After adjusting the cut-off probability the model with the same AUC = 0.88 could classify 52 of the 66 true TB cases (Table 6 and Figure 3). This however increased the number of false positive cases up to 50. The pathways that are most likely to bring in false positive cases are highlighted as broken lines (Figure 3).

**Table 6:** The logistic regression model shows the diagnostic utility of using DZM within the screening algorithm in (Figure 2).

Variable	Levels	With-out DZM			With DZM		
		Odd ratio (95% CI)	P-value	Cases classified	Odd ratio (95% CI)	P-value	Cases classified
Sample type	Sputum	1		22/66	1		33/66
	Lymph node aspirates	0.6(0.3–1.2)	0.154		0.4(0.2–0.9)	0.038	
	Abscess aspirates	4.4(1.4–14.0)	0.012		5.5(1.7–18.0)	0.004	
Smoking	No	1			1		
	Yes	2.5(1.3–4.9)	0.004		2.2(1.1–4.5)	0.027	
HIV status	Negative	1			1		
	Positive	5.4(2.3–12.5)	<0.01		5.8(2.3–14.9)	0.000	
	Unknown	0.3(0.12–0.7)	0.01		0.4(0.15–1.11)	0.081	
DZM test	Negative	–	–		1		
	Positive	–	–		16.6(5.8–47.1)	<0.001	*52/66

Area under ROC curve (AUC) = 0.843 ^without DZM^, 0.886^ with DZM^ * Case classification with adjusted cut –off probability = 0.19 at (Sensitivity = 0.81, Specificity = 0.80).

## Discussion

Direct smear microscopy (DZM) is the principal method of TB diagnosis in resource-limited settings like Mubende district, Uganda [3], [6], [32]. This is because it is a cheap procedure that can deliver results in less than a day [32]. There is a common agreement on the limitation of this test, some of which include a low sensitivity, especially on specimens with a low bacillary load [7], [8], [13]. The findings in this study are in agreement with these previous reported on DZM characteristics. For example the findings show that the greatest proportion of high bacillary load samples (+3) were identified on DZM. This means that of those that sought health care under the set criterion, DZM would most likely pick up the highly symptomatic active TB patients [21]. On the other hand, although not statistically significant, more of the low bacillary load samples (+1 and +2) were recovered by tools not used at this facility (DFM and culture).

One of the positive attributes of DZM is that it has a high specificity, and in this study a specificity of 97.1% was observed. Therefore it adequately classifies true negative cases, leaving little room for wastage of scarce resources on inappropriate treatment [32]. This limited frequency of false-positive results by DZM normally outweighs the advantages of high detection rates in mycobacterial culture considering the importance of timely diagnosis with regards to TB in resource-limited settings [5] [27]. This would not necessarily be the case for DFM [5]. On the other hand, the low kappa agreement between DZM and culture on LJ media highlights the significantly high possibility of missing of TB cases thus a low sensitivity. In this regard, DZM alone was able to correctly classify only 24 of the 66 true cases, so in the absence of a rigorous screening procedure nearly two thirds of the cases had a possibility of being missed. Studies have in the recent past documented the phenomenon of smear-negative TB cases [10], [13], [19], [33] especially among HIV infected patients. This phenomenon was also explored in this study using a logistic regression model to determine associated factors. The findings reveal that an HIV positive patient was more likely to give a false negative result on DZM than an HIV negative patient. The finding supports Corbett’s conclusion that HIV was largely associated with the increased burden of undiagnosed TB in Zimbabwe [21]. This status quo would be counterproductive to the current tuberculosis control program in Uganda, as it contributes to further spread of the disease [32]. The sample type, which was a proxy for either the patient’s pulmonary or extra-pulmonary tuberculosis status was also associated with DZM false negative results. In other words, a sample from the extra-pulmonary compartment specifically from an abscess was more likely to produce a false negative result on DZM. Although the direct link between extra-pulmonary tuberculosis and false negative results is not well documented, studies have shown that these two phenomena are associated with HIV infection [34], [35]. It should however be note that the observed association in this study is based on a small proportion of patients with abscesses, so a larger number is needed to validate the association. Smokers were on the other hand more likely to give DZM false negative result than non-smokers. Although this finding is in agreement with previous studies [36], other reports have documented it as delayed AFB conversion within smokers [37]. Analysis of drug susceptibility profiles of the DZM false negative cases shows that some of these were infected with *M. tuberculosis* bacilli which were resistant to at least one of the four drugs used as first line treatment for tuberculosis, one of them was categorized as case multi drug resistant (MDR) tuberculosis. This finding has public health implications, because among the would-be under-diagnosed group was an individual who had the potential of spreading MDR-TB to the public.

The comparison of DFM with the gold standard reveals that it has much better diagnostic characteristics than DZM, with an over 50% increment in case detection. This is in agreement with reports in Kenya [38] and India [39], although the later documented a 26% increment. On the other hand Toman’s [27] only reported a slight difference in sensitivity between the two methods but his conclusions on false positive cases between the two tools is in agreement with the current study. The higher agreement of DFM and culture on LJ media also means that more patients could get results and start treatment much faster if DFM had been used in this setting. Therefore although it would require financial and skills input, an upgrade from DZM to DFM would significantly improve the case detection especially of smear negative HIV positive TB cases at Mubende regional referral hospital. This has also been recommended elsewhere [10], [13], [38]. In reality, this diagnostic test was only carried out on a duplicate sample transported to the Mycobacteriology Laboratory, Department of Medical Microbiology, Makerere University located in Uganda’s capital city. Although DFM has been rolled out in some referral hospitals in last one year, in general it is still not widely used in health facilities in rural areas. The greatest challenge to using DFM in resource poor settings is the fact that such areas are underfunded, with over-worked personnel who receive a huge bulk of diagnostic specimens every day [11], [12]. Therefore, unless this is addressed, the diagnostic benefits of DFM could be missed if and when rolled out because the quality of the smear declines with time [27].

It would be idealistic to expect that all resource-limited settings are able to roll out programmes to use DFM in all diagnostic centres given the extra cost associated with it. For those facilities that would not immediately adopt it, the utility of a diagnostic algorithm/screening procedure with and without DZM in it was explored in this study.

WHO recommends several diagnostic algorithms for different case scenarios, however, the 2006/7 international standards for tuberculosis care is the most used [28]. The use of the clinical history like HIV status and symptoms can greatly increase the probability of detecting TB cases [40], although cases are bound to be missed if these are used in isolation [21]. The findings demonstrate that if the clinical presentation of; either cough, lymphadenitis or abscess alone was used to predict TB status, no cases would be accurately classified in this algorithm. However, a cough persistent for at least two weeks on its own has been reported to have a sensitivity of 55% among people living with HIV [8]. On the other hand in Ethiopia, it has been documented that if this type of cough were considered alone one could miss up to 56% of the cases [41]. In the current study when the first clinical presentation was combined with the patients tobacco smoking status the algorithm could accurately classify six TB cases, in effect six cases identified by this model are attributable to knowing the patients smoking status. Access to the patient’s HIV status accurately classifies another sixteen cases. To put this in context at this point the screening algorithm could accurately classify two cases less of what DZM could when used on its own. The TB diagnostic utility of the HIV status has been reported in the recent past [8], [21], [29], [42] and although there is a common recognition that HIV screening of all suspected TB cases would greatly improve case detection, it can only be adopted when simple and cheap HIV diagnostics are available in these settings [1], [21]. Adding the DZM results to this screening algorithm would accurately classify 33 of the 66 true TB cases, that is to say the model gives DZM a 13.6% (9/66) increment in case classification. The number of true cases classified was maximized to 52/66 (42% increment in case classification) by taking a specificity trade-off that inherently comes with DZM. Although this adjusted screening algorithm would improve case classification, the choice of such a screening algorithm over adopting DFM would come with a problem of over-diagnosing. This optimisation produced up to 50 false positive cases. According to estimates produced [43] on TB control in rural Uganda, if such a choice was taken in Mubende district, it could have potentially cost the control programme up to (50×351 = £17,550) in inappropriate TB management during the study period. However, the screening chart highlights the pathways that are most likely to produce these false positive cases. It is noteworthy that although the same analysis was not done with DFM as the test of choice, its diagnostics characteristics in this algorithm can be inferred from the current analysis. For example: From this analysis the gold standard gives a point of reference with regards to disease status (n = 66), therefore the three clinical predictors together with DZM give us a measure of how close we can get to detecting these 66 cases. With this in mind, if DFM alone detects 53 of the 66 cases and DZM and DFM have the same false positive rate, it was inferred that DFM would detect significantly more cases when used with the three predictors. The adjusted cut-off probability would however be different in this case. The clinical implication of this is that it is possible to arrive at an accurate diagnosis of TB with this screening algorithm using a single sample hence early treatment commencement.

Establishing bacillary load is a crucial but laborious process, which in some cases could result in ocular complications in over-worked laboratory technicians in high TB density areas [11], [12]. Therefore exploring diagnostic tool combinations that can reduce the workload is also a crucial factor in the TB control strategy. Analysis of MGIT 960 results reveals an association between time to detection and bacillary load of samples, in other words the higher the bacillary load the shorter the time to detection. This is in concordance with previous reports [17]. The use of clinical features as proxies for bacillary load shows that time to detection of bacilli was significantly associated to the following; HIV status, cigarette smoking and alcohol consumption. It might be possible to combine the clinical picture and time to detection to make inferences about bacillary load, which then eliminates the extra work put into microscopic bacillary quantification. Therefore if and when MGIT960 is introduced it is worthwhile exploring the possibility of using this technique so as to reduce the strain put on laboratory technicians in establishing bacillary load.

The limitation to this study were; 1) the number of patients used was lower than other studies that have compared diagnostics in resource limited settings. The sample size used however gave enough statistical power to detect differences. 2) a single sample per patient was used for the analysis. The WHO algorithms prescribe the use of three samples of sputum in classifying certain disease status. However, given that this was a cross sectional research study, the findings should be viewed as a cross sectional representation of the dynamics in this areas, although can be a reflection of what is happening in other resource limited settings with the same characteristics. Note that, all sputum sample collected were collected at times prescribed by WHO. Therefore the interpretations drawn from this work should take these issues in consideration.

The utility of a diagnostic test/tool/algorithm usually depends on the disease incidence in the population, the sensitivity and specificity of the test, tool and or algorithm, the physical risk and the associated cost implications. In this regard clinical symptoms are not any different from diagnostic tests with the exception of subjectivity. The challenge in clinical settings is establishing the optimum predictors (clinical questions) since the factors that govern diagnostic tools vary in populations of different geographical locale. Therefore findings in this study suggest that availing resources for screening TB suspects for HIV might be the only limiting factor in adopting such screening algorithms in resource limited settings.

## Conclusion

The study supports the concern that using DZM alone risks missing majority of TB cases, in this case we found nearly 60%, of who one was an MDR case. Although adopting DFM would reduce this proportion to 19%, the use of a three-predictor screening algorithm together with DZM was almost as good as DFM alone. It’s utility is whoever subject to HIV screening all TB suspects.

## Supporting Information

Table S1 **Questionnaire: The epidemiology of tuberculous and non-tuberculous mycobacteria in Mubende district, Uganda.**
(PDF)

Table S2 **Database: A comparison of TB diagnostic tools used on samples from Mubende regional referral hospital.**
(XLSX)
